# Successful Management of Severe Dengue With Gastrointestinal Bleeding: A Case Report Highlighting Endoscopic Hemostasis

**DOI:** 10.7759/cureus.74142

**Published:** 2024-11-21

**Authors:** Abdelrahman K Nouh, Hamza Haj Mohamad, Abduljaleel M Toubah, Abdallah A Jaber, Sana S Alkaram, Mahasin Shaheen, Ubaid Ur Rehman Hashmi

**Affiliations:** 1 Internal Medicine, Al Qassimi Hospital, Sharjah, ARE

**Keywords:** dengue fever, dengue hemorrhagic fever, endoscopic hemostasis, gastrointestinal bleeding, severe dengue

## Abstract

Dengue fever, caused by the dengue virus and transmitted by *Aedes* mosquitoes, poses a significant global health threat, particularly in tropical and subtropical regions. Severe cases can manifest as dengue hemorrhagic fever (DHF) or dengue shock syndrome, leading to complications such as plasma leakage, fluid accumulation, respiratory distress, severe bleeding, and organ impairment. Among these complications, gastrointestinal (GI) bleeding is particularly concerning due to its potential to rapidly deteriorate the patient's condition. While endoscopic hemostasis is an effective intervention for controlling GI bleeding, its application in severe dengue cases is underreported. We present the case of a male in his late 30s with severe dengue complicated by GI bleeding. Despite resuscitative measures and blood component transfusions, his condition deteriorated, necessitating endoscopic intervention for hemostasis. Successful endoscopic therapy with clips and adrenaline achieved hemostasis, highlighting the efficacy of this approach in managing severe dengue-associated GI bleeding. Primary dengue virus infection typically presents as dengue fever, while a minority progresses to develop DHF, characterized by plasma leakage and severe bleeding. Prompt recognition and management are crucial in mitigating DHF-associated morbidity and mortality. Endoscopic intervention plays a vital role in localizing and controlling bleeding sources, as demonstrated in our case. Further research is warranted to elucidate optimal treatment strategies and long-term outcomes in this patient population.

## Introduction

Dengue fever is a mosquito-borne viral infection that has a significant global health impact, particularly in tropical and subtropical regions. It is caused by one of the four dengue virus serotypes (DENV-1, DENV-2, DENV-3, and DENV-4), transmitted primarily by *Aedes *mosquitoes. Severe dengue, also known as dengue hemorrhagic fever (DHF) or dengue shock syndrome, can lead to serious complications such as plasma leakage, fluid accumulation, respiratory distress, severe bleeding, and organ impairment [1].

Among the severe complications, gastrointestinal (GI) bleeding is a critical concern due to its potential to rapidly worsen the patient's condition and lead to life-threatening situations. Endoscopic hemostasis is an effective intervention for controlling GI bleeding, yet its application in severe dengue cases remains underreported. This case report details the successful management of severe dengue with GI bleeding using endoscopic hemostasis, highlighting the challenges and strategies involved in treating such complex cases.

## Case presentation

A male in his late 30s with no significant past medical history presented with a chief complaint of melena, hematochezia, and hematemesis. The patient's condition began six days prior with a fever that persisted for four days. Then, two days before admission, he noticed black, tarry stools and occasional fresh blood in his stools. He also experienced an episode of vomiting blood. He reported living in an area with a significant increase in mosquito activity following recent heavy rains and experiencing multiple mosquito bites.

On initial evaluation, he was alert, conscious, and oriented but appeared pale and ill. His vital signs were notable for a heart rate of 126 beats per minute (bpm), a respiratory rate of 17 breaths per minute, a blood pressure of 80/35 mmHg, and an oxygen saturation of 100% on room air. Physical examination revealed a soft and non-tender abdomen and clear chest sounds. There were no neurological deficits.

Initial management in the emergency department included the administration of 2 L of intravenous fluids and one unit of O-negative packed red blood cells (PRBC), with two additional units arranged. An infusion of pantoprazole was started, along with intravenous ceftriaxone. The patient was admitted to the intensive care unit (ICU) for close monitoring and further management. Upon initial evaluation, the patient's complete blood count (CBC) showed significant abnormalities. Table 1 shows the laboratory tests on admission.

**Table 1:** Laboratory test results during admission

Parameter	Result	Normal Range	Interpretation
Hemoglobin (g/dL)	9.90	13.00-17.00	Anemia
Hematocrit (%)	27.80	40.00-50.00	Anemia
White blood cell (WBC) (×10^3^/mcL)	5.80	4.00-11.00	Normal
Platelet count (×10^3^/mcL)	107.00	150.00-450.00	Thrombocytopenia
Prothrombin time (PT)	12	11-13.5 seconds	Normal coagulation profile
Partial thromboplastin time (PTT)	29	25-35 seconds	Normal coagulation profile
INR (international normalized ratio)	0.9	0.8-1.1	Normal coagulation profile
Total protein (g/L)	45.0	64-82	Significantly low
Albumin (g/L)	22.0	34.0-50.0	Significantly low
AST (aspartate aminotransferase) (U/L)	40	15-37	Elevated
C-reactive protein (CRP) (mg/L)	14.3	0.0-3.0	Markedly elevated
Dengue virus PCR	Detected	Not detected	Dengue hemorrhagic fever confirmed

Despite initial resuscitative measures, the patient's melena persisted. An additional liter of intravenous fluids was administered, and a second unit of PRBC was transfused. A repeat CBC conducted five hours after ICU admission revealed a further decrease in platelet count to 72.00x10^3^/mcL, and hematocrit dropped to 20.00%, accompanied by a decline in WBC to 3.24x10^3^/mcL. Given the rapid decline in platelet counts and ongoing bleeding, a platelet transfusion was initiated. The patient's blood pressure remained critically low at 75/30 mmHg, necessitating the initiation of norepinephrine for hemodynamic support. Ivabradine was given to the patient to manage tachycardia, which had escalated to 140 bpm.

**Figure 1:**
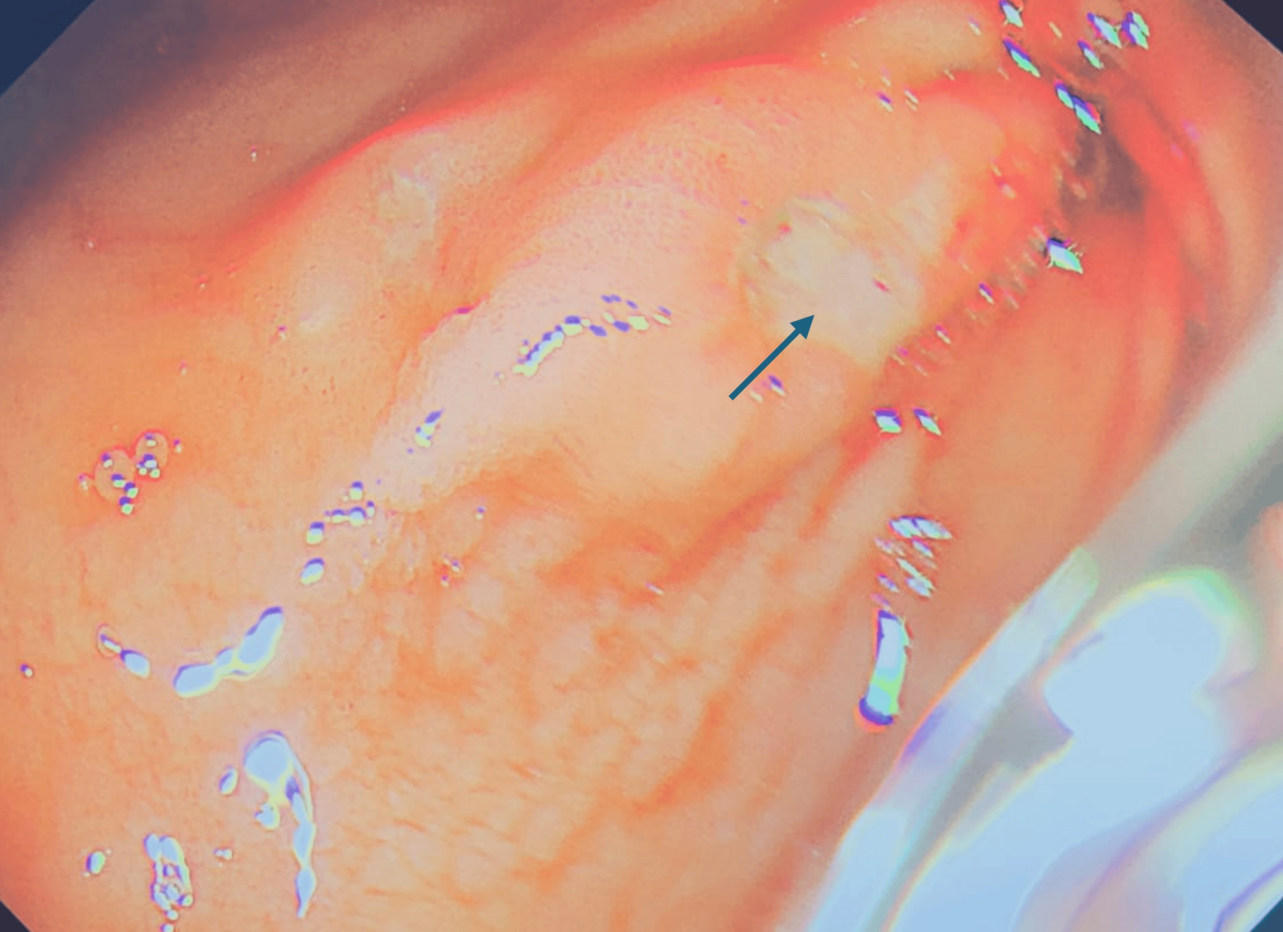
Clean-based ulcers in the antrum, marked by the arrow.

**Figure 2:**
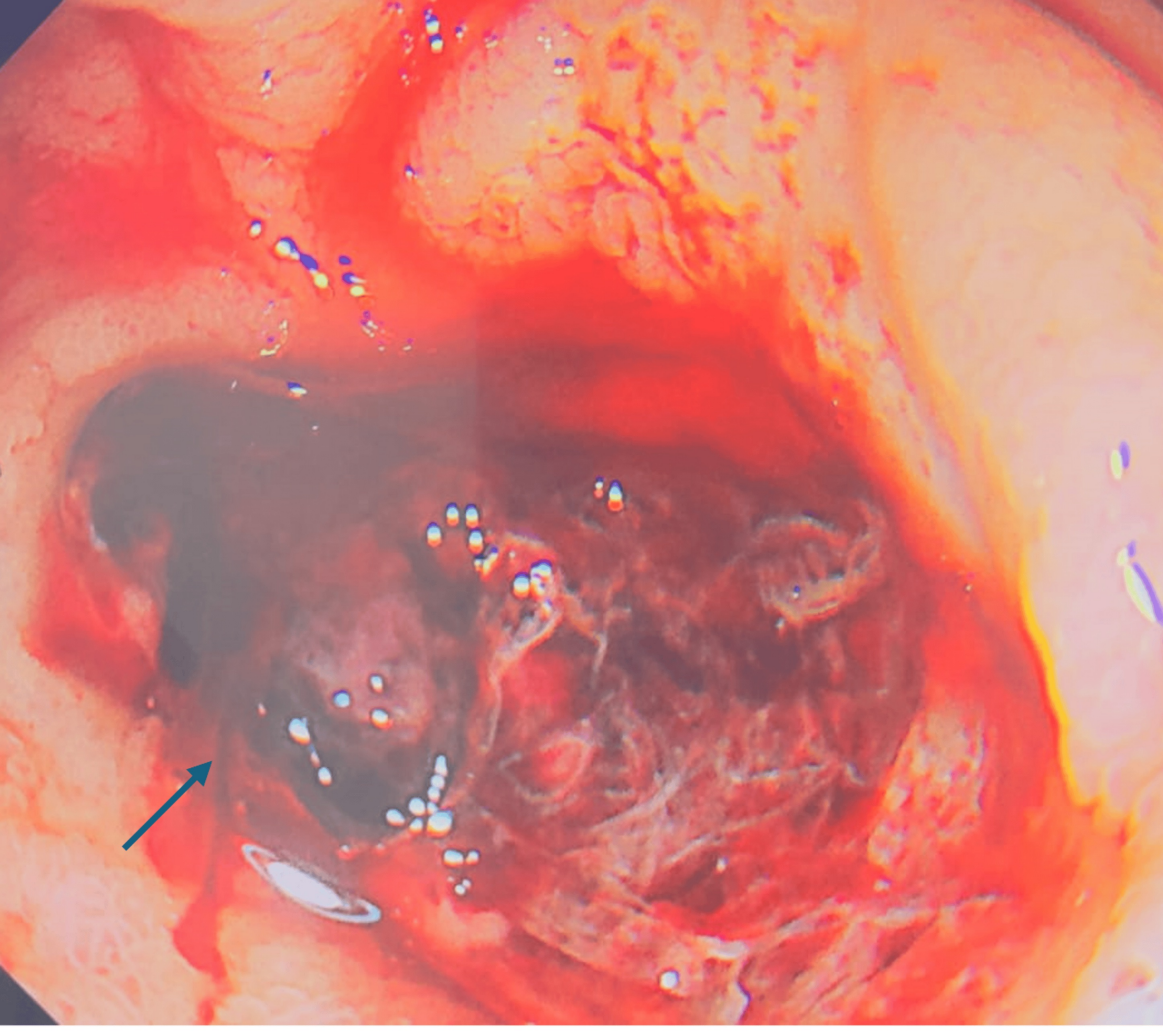
Duodenal ulcer with an adherent clot, marked by the arrow.

**Figure 3:**
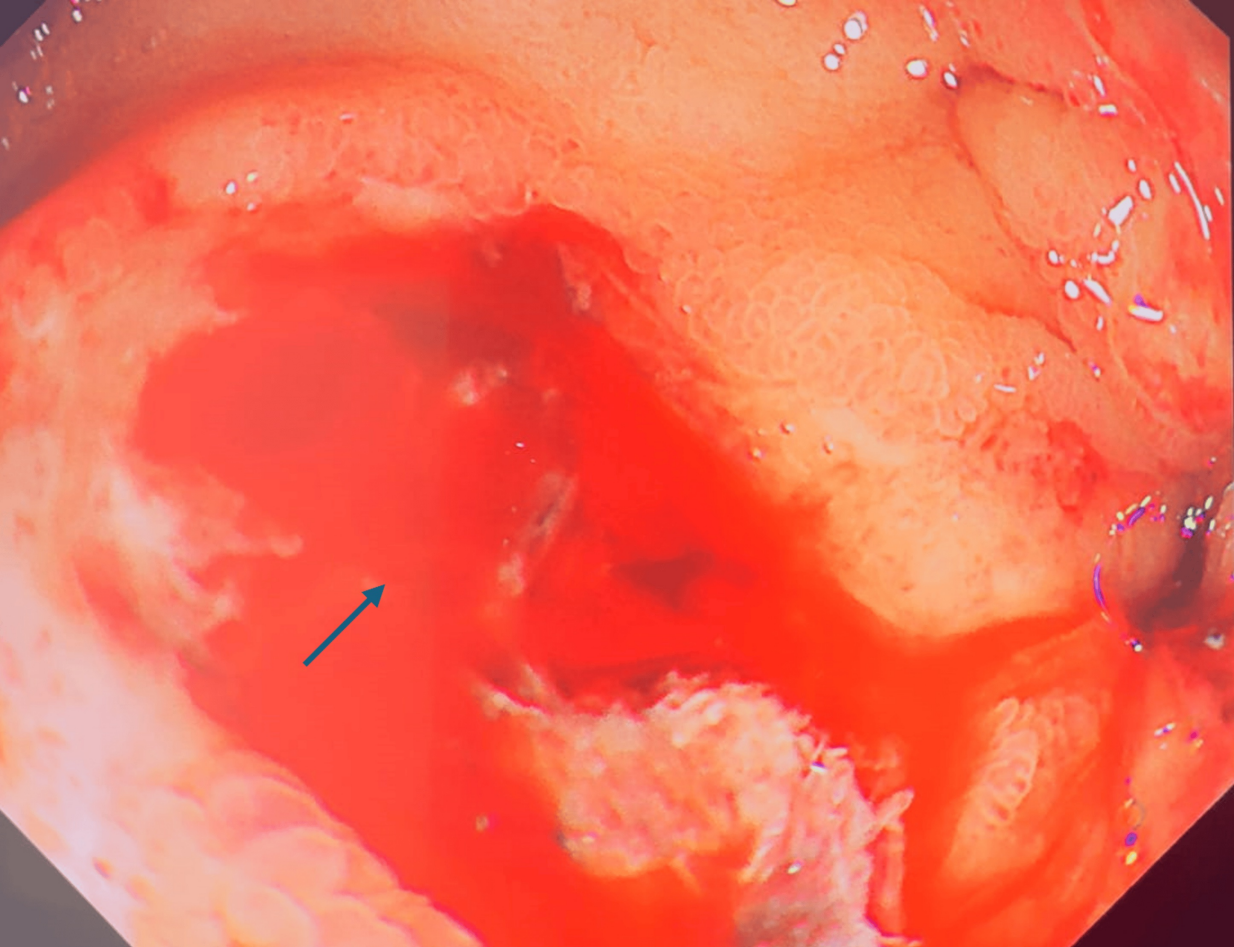
Active ooze from the base of the ulcer (Forrest 1B) after removing the clot, marked by the arrow.

**Figure 4:**
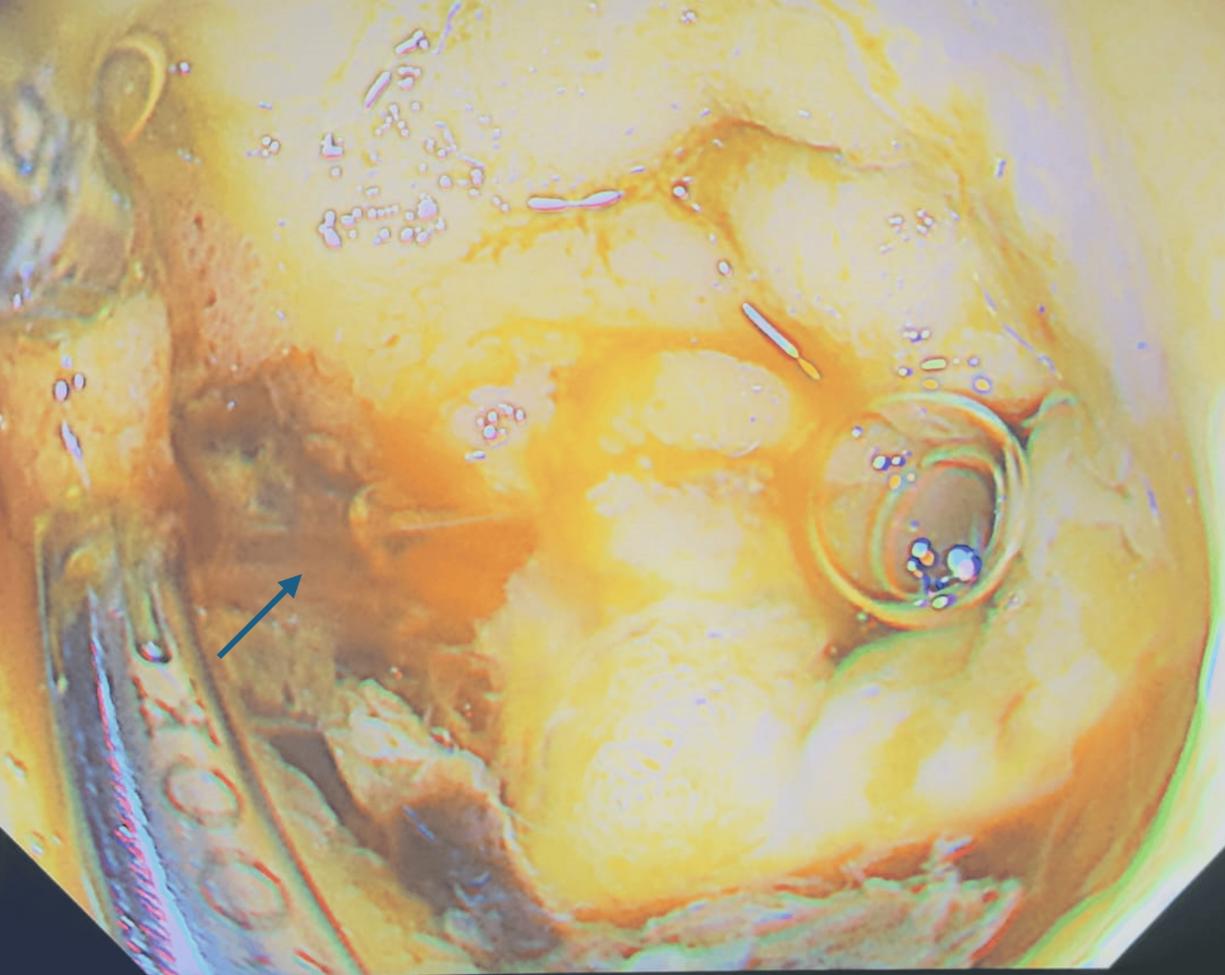
Hemostasis was achieved with hemoclips and an adrenaline injection, marked by the arrow.

Given the lack of improvement with current management, it was decided that the patient would undergo an esophagogastroduodenoscopy (EGD) procedure to control the ongoing active bleeding. During the procedure, the patient was positioned in the left lateral decubitus position following a 12-hour fasting and pre-medication with Xylocaine spray 10% for pharyngeal anesthesia and IV Midazolam for sedation. Continuous monitoring of vital signs ensured patient safety throughout. Esophageal examination revealed a Z line at 36 cm with Hill grade 1 and a normal mucosal appearance. Notably, the stomach displayed unremarkable mucosa in the fundus and body regions but harbored two sizable, clean-based ulcers in the antrum. Furthermore, the duodenal assessment uncovered a Forrest 1B oozing hemorrhage in D1, which was successfully managed using endotherapy with clips and adrenaline, achieving hemostasis (Figures 1-4). In anticipation of potential recurrent bleeding, plans were devised for further imaging with computer tomography (CT) angiography and interventional radiology embolization.

CT angiography was done and revealed an unremarkable study, and as the patient was not actively bleeding, the team opted against proceeding with interventional radiology embolization. The patient was kept in the ICU for observation and management.

The patient initially experienced clinical deterioration, evidenced by a significant drop in hemoglobin to 5.6 g/dL, platelets to <9.5x10^3^/mcL, and elevated liver enzymes. In response, Gastroenterology recommended close monitoring for ongoing bleeding and considered angiography and embolization if necessary. Interventional Radiology initiated arterial line insertion and planned embolization; however, this was postponed due to operational constraints. Nevertheless, the patient stabilized without active bleeding, leading to transfer from the ICU to the ward for continued monitoring and supportive care. During this period, the patient received a total of 4 units of PRBCs, 1 unit of platelets, and 1 unit of fresh frozen plasma (FFP). Subsequently, the patient exhibited significant clinical improvement, with the resolution of melena and other active complaints. Notably, hemoglobin levels stabilized, and there was a notable improvement in platelet count. Upon discharge, the patient remained on pantoprazole and was scheduled for a follow-up appointment after two weeks, during which total improvement and the absence of complaints were observed.

## Discussion

Primary infection with the dengue virus is more likely to manifest as dengue fever, characterized by fever and a range of non-specific symptoms such as headache, retroorbital pain, myalgia, and, occasionally, hemorrhagic symptoms. A minority of individuals progress to develop DHF, the most severe form of the disease. DHF is characterized by plasma leakage, leading to intravascular volume loss and circulatory insufficiency. Severe bleeding is another prominent feature associated with severe cases. While bleeding is common in both dengue fever and DHF, GI bleeding is more prevalent in DHF cases. Additionally, elevated liver enzyme levels and thrombocytopenia are frequently observed in both dengue fever and DHF cases, with greater severity typically seen in DHF cases [2]. The presented case underscores the clinical challenges and management strategies in DHF complicated by massive GI bleeding.

Prompt recognition and management are paramount in mitigating the morbidity and mortality associated with DHF. Initial resuscitative measures, including intravenous fluid resuscitation and blood component transfusions, aim to stabilize hemodynamic parameters and correct coagulopathy [3]. The administration of pantoprazole and antibiotics serves to prevent stress ulceration and address potential bacterial superinfection, respectively, given the heightened risk in critically ill patients [4]. Endoscopic intervention plays a crucial role in localizing and controlling bleeding sources in DHF-associated GI hemorrhage. Our patient underwent successful endoscopic hemostasis with clips and adrenaline injections, highlighting the effectiveness of this approach in achieving hemostasis and preventing recurrent bleeding. However, the decision to pursue endoscopic therapy should be balanced with the patient's clinical status, bleeding severity, and procedural risks [5]. The role of adjunctive therapies, such as vasoactive agents, remains a subject of debate in DHF management [3]. In this case, the initiation of norepinephrine and ivabradine aimed to address hemodynamic instability and tachycardia, respectively, although their impact on clinical outcomes warrants further investigation.

IR techniques, including angiography and embolization, represent valuable tools in managing refractory bleeding in DHF. Although IR was considered in our patient, operational constraints precluded its implementation. Nonetheless, the absence of active bleeding and the stabilization of hemoglobin levels following supportive care underscore the importance of a multidisciplinary approach in optimizing patient outcomes.

## Conclusions

This case contributes to the existing literature on DHF-associated GI bleeding by highlighting the efficacy of endoscopic intervention and the challenges in implementing adjunctive therapies. Further research is warranted to elucidate optimal management strategies, particularly regarding the role of adjunctive pharmacotherapy and the timing of interventional procedures in DHF-associated bleeding. Additionally, prospective studies are needed to evaluate the long-term outcomes and complications associated with different treatment modalities in this patient population.
